# Investigation of standardized ileal amino acid digestibility of rice protein meal in sexed broilers

**DOI:** 10.1016/j.psj.2025.105075

**Published:** 2025-03-19

**Authors:** Muhammad A Iqbal, Tanveer Ahmad, Muhammad S Ahmad, Usama Aftab, Nasir Mukhtar

**Affiliations:** a Department of Livestock Production and Management, PMAS Arid Agriculture University, Rawalpindi, Pakistan; b University Institute of Biochemistry and Biotechnology, PMAS Arid Agriculture University, Rawalpindi, Pakistan; c AB Vista PTE Ltd., Singapore

**Keywords:** Broiler, Digestibility, Rice protein meal, Amino acid, Protease

## Abstract

Rice protein meal (**RPM**), a byproduct of the rice industry, has emerged as a potential alternative for poultry feed, especially in rice-producing regions in the South Asia region. The aim of this study was to evaluate the standardized ileal amino acid (**AA**) digestibility of RPM produced from two distinct rice varieties in sexed broiler chickens, with or without the inclusion of a commercial protease enzyme. Treatments were structured in a 2 × 2 × 2 factorial arrangement, including two sources of RPM (Basmati and IRI), sex (male and female), and protease levels (0 and 500 units per ton of feed). In addition to the test diets, a reference diet based on soybean meal (SBM) and a nitrogen-free diet (**NFD**) were included in the experiment. Titanium dioxide was added on 0.30% of the diets as an inert marker. Experimental diets were fed to 6 replicates, each consisting of male and female chicks, from 21 to 28 days of age. The data were analyzed using General Linear Model techniques with the help of SPSS 16.0. The main effect of varieties was significant (*P* < 0.05), as the standardized ileal digestibility (**SID**) of lysine (57.8%) and threonine (50.7%) was higher for Basmati compared to IRI. The SID for arginine was lower (*P* = 0.02), and the SID of cysteine (14.4%), isoleucine (47.3%), leucine (46.1%), valine (45.2%), and histidine (49.6%) tended to be lower (*P* < 0.07) in females for RPM derived from Basmati rice. Protease had no effect on the SID of AA, nor did any of the other three- or two-way interactions.

## Introduction

Traditionally, broiler diets have relied heavily on conventional protein sources such as soybean meal (**SBM**), (Vlaicu et al., 2024). However, increasing demand, fluctuating prices, and concerns regarding environmental sustainability, have necessitated the exploration of alternative protein sources (Dinani, et al., 2020). True value of amino acid (**AA**) digestibility is crucial for cost-effective feed formulation and efficient utilization of feed materials. A system based on digestible AA is clearly superior to one based on total AA for expressing the amounts available for maintenance and production purposes (Lemme, et al., 2004). The concept of digestible AA values is increasingly recognized as a useful tool in poultry feed formulations (Azam, et al., 2019). In modern poultry production, achieving optimal growth performance and feed efficiency is essential for ensuring economic viability and sustainability. Evaluating the true value of protein in indigenous protein sources is, therefore, a key area of research interest. Central to this objective is the formulation of nutritionally balanced diets that precisely meet the requirements of growing broilers.

The best estimates of the AA digestibility in poultry were based on analyzing digesta collected from the ileum, as it reflects the proportion available to the animal for metabolism (Ravindrana, et al., 2017). The assessment of the digestible AA becomes more important for feed ingredients with inherently low digestibility values (Sultan, et al., 2024). The most crucial steps in performing a digestibility trial include formulating a feed with the only source of nitrogen from the tested ingredient, proper mixing, *ad-libitum* feeding for a specific period, slaughtering or euthanasia of birds, collection of digesta form the terminal ileum, and finally the analysis of test ingredients, assay diets and ileal digesta. Minor variation in any of these steps can affect the values obtained for AA digestibility.

This research aimed at a systematic evaluation of the AA digestibility of rice protein meal (**RPM**) in sexed broilers. RPM is a byproduct of rice starch or rice syrup production, primarily derived from broken rice. It is a plant-based protein source commonly used in animal feed and sometimes in human nutrition. Whole rice grains or broken rice are cleaned, milled and treated with enzymes or water to extract starch, leaving behind a protein-rich fraction. The protein is separated from fibers and residual starch through centrifugation or filtration. The wet protein mass is dried and ground into a fine meal or powder. RPM is valued for its high protein content (40-60%), making it a good alternative protein source in animal feed, aquaculture, and even plant-based diets. By employing robust methodologies and experimental designs, this study seeks to provide valuable insights into the nutritional value of RPM and its potential as an alternative protein source in broiler diets. The findings of current research are significant for optimizing diet formulations based on digestible AA, which ultimately enhances feed efficiency and promotes sustainable poultry feeding practices.

## Materials & methods

This study was conducted in accordance with the ethical guidelines set by the Institutional Ethic Committee of PMAS Arid Agriculture University, Rawalpindi, Pakistan. The research protocol was reviewed and approved following the university's standard ethical procedures for animal research. An application process was followed, including submission of study details, and oversight review ensured compliance with ethical standards for the care and use of broilers in research.

### Birds and housing

The current experiment was conducted at Research and Development center, Sultan Feed Mills, Sargodha, Punjab, Pakistan with collaboration of the PMAS ARID Agriculture University, Rawalpindi, Pakistan. The main ingredient used in this study was RPM, representing two different varieties of rice (Basmati and IRI) from two different regions of Pakistan. A diet based on SBM was included as a reference standard. The test ingredients served as the only source of nitrogen in the assay diets.

A total of 720-day-old broiler chicks (male and female) were procured from a commercial hatchery and divided into 11 distinct experimental groups. 8 treatment groups (8 × 6*10) having total of 480 sexed birds, under a completely randomized design in a three-way factorial arrangement. Main factors included two varieties of rice protein meal (IRI and Basmati), sex (male and female), and protease levels (0 and 500 units/t of feed). Each group had 6 replicates, with 10 sexed birds per replicate. 2 sperate dietary groups were used to accumulate endogenous losses in male and female birds by providing nitrogen free diet and 11th group with SBM-based diet was used as reference group for cross verification of methodology and procedure in mixed birds. Chicks were kept and reared under controlled environmental conditions. Temperature was controlled at 33°C from the first day and subsequently reduced to 26°C till the 28th day. Continuous light and fresh water were supplied within the floor pens. Birds were offered a commercial starter diet containing 23% crude protein (Aviagen, 2022) from day 1 to 12, and grower diet containing 21.5% crude protein from day 13 to 21 days. On day 22, test diets were offered *ad-libitum* to all the chicks which were distributed accordingly.

### Experimental diets

Test diets were formulated with RPM as the only source of nitrogen, resulting in a net crude protein level of 20% in the final feed. Additionally, NFD was formulated to account for endogenous losses (Table 1). The inclusion levels of RPM in the diet were adjusted on a crude protein (CP) basis to ensure that the dietary CP level met the breed standard (Ravindran and Abdollahi, 2021). Corn starch and dextrose were also used as energy sources in these diets. Titanium dioxide, an inert marker, was added at 0.3% to each diet (Ravindran, et al., 2017) from day 21 to 28 to determine the standardized ileal digestibility (**SID**). A NFD was formulated to determine endogenous AA losses to calculate SID (Adedokun, et al., 2007). Test diets were formulated with and without supplemental protease enzyme (VTR BioTech at 500 g per ton of feed) to check the effect on digestibility.

**Table 1:** Experimental diets for standardized ileal digestibility trial of rice protein meal (RPM), reference diet i.e. soybean meal (SBM) and nitrogen free diet (NFD).

Ingredient (%)	Diet 1 Basmati	Diet 2 IRI	Diet 3 SBM^4^	Diet 4 NFD^5^
Rice protein meal	45.0	35.0		
Soybean meal			40.0	
Dextrose	23.0	28.0	26.0	44.0
Arbocel^1^	-	-	-	3.0
Corn starch	23.2	28.2	25.2	43.2
Soya oil	4.0	4.0	4.0	5.0
Dicalcium phosphate 18%	1.9	1.9	1.9	1.9
Limestone	1.3	1.3	1.3	1.3
Sodium bicarbonate	0.3	0.3	0.3	0.3
Sodium chloride	0.3	0.3	0.3	0.3
Choline chloride	0.3	0.3	0.3	0.3
Vitamin premix^2^	0.2	0.2	0.2	0.2
Mineral premix^3^	0.2	0.2	0.2	0.2
Titanium dioxide	0.3	0.3	0.3	0.3
Total	100.0	100.0	100.0	100.0

1 Arbocel, Insoluble raw fiber concentrate, Holzmuhle, Rosenberg, Germany.

2 Provided per kg of diet: retinyl acetate, 4,500 IU; cholecalciferol, 120 μg; DL-α-tocopherol acetate, 12 IU; menadione sodium bisulphite, 2.50 mg; thiamine, 2.5 mg; riboflavin, 5.0 mg; niacin, 32 mg; d-pantothenic acid, 10 mg; pyridoxine, 5 mg; biotin, 132 μg; folic acid, 2.5 mg; cyanocobalamin, 20 μg.

3 Provided per kg of diet: manganese, 90 mg (MnSO4.H2O); iron, 85 mg (FeSO4.H2O); zinc, 80 mg (ZnO); copper, 5 mg (CuSO4.5H2O); iodine, 1 mg (ethylene diamine dihydroiodide); selenium, 125 μg (Na2SeO3).

4 SBM = soybean meal.

5 NFD = nitrogen free diet.

### Sampling procedure

On day 29, 6 male and 6 female birds from each replicate were slaughtered (Azam, et al., 2019) to collect digesta from the terminal ileum, defined as the portion of the small intestine starting from the Meckel's diverticulum to 40 mm proximal to the ileo-cecal junction (Bandegan, et al., 2010) by gently flushed with distilled water. Digesta was immediately stored at −20°C, followed by freeze drying (Kong and Adeola, 2014). Dried samples were then ground to pass through a 0.5 mm sieve and stored in plastic bags at −4°C for further analyses (Bandegan, et al., 2010).

### Chemical analyses & calculations

Test ingredients, assay diets, and dried ileal digesta samples for each replicate were analyzed for total AA contents at Masters Lab, Netherlands, (accredited by the RvA Dutch Accreditation Council L172). Proteins were hydrolyzed using 6N HCl at 110°C for 24 h under an inert atmosphere. The resulting amino acids were derivatized and analyzed using Reversed-phase (RP-HPLC) with a C18 column and fluorescence detection (Bartolomeo and Maisano, 2006). Samples of test ingredients, test diets and collected digesta were analyzed with separate method (AOAC., 2006), for dry matter (AOAC, 2006 method 930.15), ether extract (AOAC, 2006 method 920.39), crude fibre (AOAC, 2006 method 978.10), CP (*N ×* 6.25) (AOAC, 2006 method 994.13) and ash contents (AOAC, 2006 method 942.05). Titanium dioxide in all test diets and digesta samples were analyzed with UV spectrophotometer, as described by (Morgan, et al., 2014). The data were analyzed by the General Linear Model (**GLM**) using multivariate analysis of SPSS 16.0. Differences were considered significant at *(P < 0.05)*. The endogenous amino acid (**EAA**) concentration was calculated as milligrams of AA flow per kg of dry matter intake (**DMI**).

Ileal AA flow (mg/kg) DMI = [AA in ileal digesta (mg/kg) × (Diet marker (mg/kg) / Ileal marker (mg/kg)]

The apparent ileal digestibility coefficient of AA was calculated as shown below:

AIDC = (AA/Ti) diet – (AA/Ti) Ileal /(AA/Ti) diet

(AA/Ti)_Diet_ = Ratio of AA to Titanium in Diet, (AA/Ti)_ileum_=Ratio of AA to Titanium in Digetsa

Data obtained from Apparent ileal digestibility were converted to SID by basal endogenous AA values. The endogenous ileal AA losses were used to calculate SID by the following equation.

SID (%) = AID + (Basal EAA */* Ingredient AA) *×* 100

AID = Apparent ileal digestibility, EAA = Endogenous amino acids, Ti = Titanium

## Results

### Proximate and total amino acids

The analyzed proximate composition and total AA content of the two varieties (Basmati and IRI) and SBM are presented in Table 2. In both varieties, leucine, arginine, and valine were the most prominent AA. The AAs appearing in the least amount in both varieties were cysteine, followed by histidine. Except for lysine, AA concentration was proportional to CP in RPM. Proximate and total AA concentration of SBM were in line with the published data (Ullah, et al., 2016).

**Table 2:** Analyzed proximate and total amino acid (TAA) composition (%) of assay diets and test ingredients, as-fed basis.

Nutrient (%)	Diet 1^1^	Diet 2^2^	Diet 3^3^	Basmati	IRI	SBM^4^
Moisture	12.10	11.20	10.50	5.90	5.20	10.10
Dry matter	87.90	89.80	89.50	94.10	94.80	89.90
Crude protein	21.22	20.34	20.50	41.13	50.97	45.80
Crude fat	7.00	6.00	5.63	4.00	2.50	1.98
Crude fiber	1.57	1.05	1.75	3.50	0.30	3.95
Ash	4.50	4.50	3.50	9.00	5.00	6.55
Lysine	0.68	0.52	1.12	1.58	1.57	2.80
Methionine	0.55	0.51	0.25	1.28	1.52	0.62
Cysteine	0.35	0.38	0.26	0.81	1.15	0.66
Threonine	0.74	0.67	0.72	1.73	2.07	1.79
Arginine	1.43	1.49	1.33	3.33	4.45	3.34
Isoleucine	0.84	0.81	0.84	1.95	2.41	2.10
Leucine	1.57	1.57	1.40	3.66	4.70	3.50
Glycine	0.91	0.85	0.78	2.12	2.55	1.95
Valine	1.08	1.09	0.88	2.52	3.26	2.20
Histidine	0.43	0.42	0.48	0.99	1.25	1.21

1 Diet containing Basmati variety of rice protein meal.

2 Diet containing IRI variety of rice protein meal.

3 Diet containing soybean meal as the reference ingredient.

4 SBM = soybean meal.

### Standardized Ileal digestibility (SID)

#### Main effects

The main effects of variety, sex and protease are presented in Table 3. SID of AA between varieties revealed significant difference (*P* < 0.05) for lysine and threonine. Basmati had a higher SID for lysine and threonine compared to IRI. The highest SID value was observed for arginine in IRI, followed by lysine in Basmati, while the lowest SID was observed for cysteine in Basmati, followed by methionine in both varieties. The SID of AA between sex also revealed significant differences (*P* < 0.05) for lysine, cysteine, threonine, glycine, valine and histidine. Male broilers had higher SID values for lysine, cysteine, threonine, glycine, valine and histidine, while the SID of methionine, arginine, isoleucine and leucine did not reveal any notable differences (*P* > 0.05). The highest SID values were for arginine, followed by lysine were observed in male broilers, while the lowest SID was observed in cysteine. The highest SID in female broilers was observed for arginine, followed by histidine, while the lowest value was determined for cysteine. No significant differences (*P* > 0.05) were observed in SID values with protease supplementation. The highest SID value without protease supplementation was observed for arginine, followed by histidine, while the lowest SID was observed for cysteine. The highest SID value when supplemented with protease was observed for arginine, followed by histidine, while the lowest SID was observed for cysteine.

**Table 3:** Standardized ileal digestibility (SID) of amino acids for soybean meal (SBM), varieties, sex and protease supplementation (one-way effect)^1^.

Treatments			Parameters^2^									
SID			Lys	Met	Cys	Thr	Arg	Ile	Leu	Gly	Val	His
Soybean Meal (SBM)			89.1	88.8	76.6	84.2	92.3	87.3	86.6	87.8	85.9	89.4
Variety^3^	Sex^4^	Protease										
V1			57.8^a^	40.5	21.2	50.7^a^	71.4	50.6	49.4	50.3	48.7	52.9
V2			46.5^b^	40.2	24.4	46.9^b^	72.9	50.8	51.3	48.6	50.4	53.6
SEM			1.2	1.7	1.9	1.1	0.7	1.1	1.1	0.9	1.1	1.0
P Value			0.00	0.91	0.24	0.03	0.12	0.89	0.25	0.24	0.29	0.64
	M		54.9^a^	41.8	27.0^a^	51.8^a^	72.8	52.3	51.9	52.3^a^	51.3^a^	54.9^a^
	FM		49.4^b^	38.9	18.6^b^	45.7^b^	71.4	49.0	48.7	46.6^b^	47.8^b^	51.5^b^
	SEM		1.2	1.7	1.9	1.1	0.7	1.1	1.1	0.9	1.1	1.0
P Value			0.00	0.24	0.01	0.00	0.16	0.06	0.07	0.00	0.04	0.03
		No	51.3	41.1	22.7	49.1	72.3	50.6	50.5	48.8	49.9	53.1
		YES	53.0	39.6	22.9	48.4	72.0	50.8	50.2	50.1	49.2	53.4
SEM			1.2	1.7	1.9	1.1	0.7	1.1	1.1	0.9	1.1	1.0
P Value			0.29	0.55	0.95	0.67	0.79	0.89	0.83	0.36	0.71	0.86

1 Values in the same row with different superscripts are significantly different *(P<0.05)*.

2 Parameters Lys = lysine, Met = methionine. Cys = cysteine, Thr = threonine, Arg = arginine, Ile = isoleucine, Leu = leucine, Gly = glycine, Valine = valine, His = histidine.

3 Varieity, V1 = Basmati, V2 = IRI.

4 Sex, *M* = Male, FM = female.

#### Two-way effect

The mean SID of AA for two-way interaction is presented in Table 4. The SID of arginine was lower (*P* = 0.02), and those of cysteine, isoleucine, leucine, valine, and histidine tended to be lower (*P* < 0.07) in female broilers for RPM derived from Basmati rice. The highest SID values were determined for arginine in IRI variety fed to male broilers, followed by lysine in the same variety and sex of the broiler. The lowest SID was determined for cysteine in the Basmati variety fed to female broilers. The interaction between varieties and protease supplementation also revealed that there were no differences (*P* > 0.05). Similarly, the interaction between sex and protease supplementation also observed no difference (*P* > 0.05).

**Table 4:** Standardize ileal digestibility (SID) for amino acids (AA) of rice protein meal (RPM), variety x sex, variety x protease and sex x protease (two-way effect)^1^.

Treatment			Parameters^2^									
Variety^3^	Sex^4^	Protease	Lys	Met	Cys	Thr	Arg	Ile	Leu	Gly	Val	His
V1	M		61.8	43.7	28.0	55.0	73.3^a^	53.8	52.6	54.1	52.1	56.2
	FM		53.7	37.3	14.4	46.3	69.4^c^	47.3	46.1	46.5	45.2	49.6
V2	No		48.0	39.9	26.0	48.7	72.4^b^	50.9	51.3	50.6	50.6	53.7
	YES		45.0	40.5	22.8	45.0	73.5^a^	50.7	51.3	46.7	50.3	53.5
SEM			1.6	2.4	2.7	1.6	1.0	1.6	1.6	1.3	1.6	1.4
P Value			0.14	0.17	0.07	0.13	0.02	0.06	0.06	0.19	0.05	0.05
V1		No	57.5	42.8	22.9	51.2	71.9	51.4	50.7	49.8	50.1	53.4
		YES	58.1	38.3	19.5	50.1	70.8	49.7	48.0	50.7	47.2	52.4
V2		No	45.1	39.4	22.6	46.9	72.6	49.7	50.3	47.8	49.6	52.8
		YES	48.0	41.0	26.3	46.8	73.2	51.9	52.3	49.4	51.3	54.3
SEM			1.6	2.4	2.7	1.6	1.0	1.6	1.6	1.3	1.6	1.4
P Value			0.48	0.23	0.20	0.75	0.38	0.24	0.16	0.79	0.17	0.41
	M	NO	54.0	42.1	26.6	51.8	72.8	51.7	51.5	51.2	51.0	54.8
		YES	55.8	41.6	27.4	51.8	72.9	52.9	52.3	53.5	51.7	55.0
	FM	NO	48.5	40.1	18.9	46.3	71.7	49.4	49.5	46.5	48.7	51.4
		YES	50.2	37.7	18.4	45.0	71.1	48.6	48.0	46.7	46.8	51.7
SEM			1.6	2.4	2.7	1.6	1.0	1.6	1.6	1.3	1.6	1.4
P-Value			0.97	0.71	0.79	0.67	0.72	0.55	0.49	0.46	0.43	0.98

1 Values in the same row with different superscripts are significantly different *(P<0.05)*.

2 Parameters Lys = lysine, Met = methionine. Cys = cysteine, Thr = threonine, Arg = arginine, Ile = isoleucine, Leu = leucine, Gly = glycine, Valine = valine, His = histidine.

3 Varieity; V1 = Basmati, V2 = IRI.

4 Sex; *M* = Male, FM = female.

#### Three-way effects

The three-way interaction (variety × sex × protease) was non-significant (*P* > 0.05) for any of the selected traits (Table 5). In case of reference diet, the average SID of different AA was almost identical to the published data of (Ullah, et al., 2016).

**Table 5:** Standardize ileal digestibility (SID) for amino acids (AA) of rice protein meal (RPM), varieties x sex x protease (three-way effect)^1^.

Treatments			Parameters^2^									
Variety^3^	Sex^4^	Protease	Lys	Met	Cys	Thr	Arg	Ile	Leu	Gly	Val	His
V1	M	NO	60.4	44.2	27.9	54.4	73.1	53.1	52.3	51.9	51.8	55.9
		YES	63.3	43.3	28.1	55.6	73.5	54.5	52.9	56.2	52.5	56.4
	FM	NO	54.6	41.4	17.9	48.1	70.7	49.7	49.2	47.8	48.5	50.8
		YES	52.9	33.2	10.9	44.5	68.1	44.9	43.1	45.2	42.0	48.4
V2	M	NO	47.6	40.1	25.3	49.3	72.5	50.3	50.8	50.5	50.2	53.7
		YES	48.4	39.8	26.8	48.1	72.3	51.4	51.7	50.7	50.9	53.7
	FM	NO	42.5	38.8	19.9	44.6	72.8	49.1	49.8	45.2	49.0	52.0
		YES	47.6	42.2	25.8	45.5	74.1	52.4	52.9	48.2	51.6	55.0
SEM			2.3	3.4	3.8	2.2	1.4	2.3	2.3	1.9	2.3	2.0
P-Value			0.19	0.27	0.29	0.29	0.26	0.22	0.18	0.09	0.17	0.33

1 Values in the same row with different superscripts are significantly different *(P<0.05)*.

2 Parameters Lys = lysine, Met = methionine. Cys = cysteine, Thr = threonine, Arg = arginine, Ile = isoleucine, Leu = leucine, Gly = glycine, Valine = valine, His = histidine.

3 Varieity; V1 = Basmati, V2 = IRI.

4 Sex; *M* = Male, FM = female.

## Discussion

The objective of this study was to determine the digestible AA content in RPM. This information is pivotal to recommend a new ingredient as alternative feed source in broiler diets (Salehifar, et al., 2012). Rice is considered an important cereal due to its high-quality protein (Jayaprakash, et al., 2022). Using rice protein meal in poultry diets is a relatively new concept, further restricted by the lack of published data on digestible AA values for broilers (Wani, et al., 2018). Previous study was conducted to evaluate the effects of using different inclusion levels of RPM in broilers (Wani, et al., 2018). But this study does not provide the AA digestibility data to utilize in least cost feed formulation with ideal AA balanced diet. RPM is a byproduct of the rice starch industry (Dinani, et al., 2020). Rice protein meals obtained from this process may have lower or variable commercial importance, primarily due to their lower quality or poor nutritional value. RPM contains highly insoluble proteins due to disulfide linkages and also has very high molecular weights of naturally occurring proteins like glutelin (Souza, et al., 2017). Moreover, it has been widely proven that some major proteins in rice exhibit vital biological activities (Jayaprakash, et al., 2022), underscoring their importance as valuable feed or food materials (Mohammed K. W., 2018). The process of starch removal by enzymatic hydrolysis has been widely used, but it may affect the quality of final product if the specific parameters during the procedure are not controlled or monitored. Enzymatic hydrolysis is the most effective, secure, and most common method for the modification of plant protein (Souza, et al., 2017). Removal of starch from a grain undergoes several factors which may result in variable quality of final product. These factors include temperature, enzyme concentration and incubation time. Any minute changes in the set parameters will not only result in variable contents of CP in residual material but also affect the quality of protein fractions as well.

The results of the current study revealed that the SID of AA was lower in RPM compared to SBM. A SBM based reference diet was included in the study to ensure that factors such as drying, marker and mixability were well-controlled (Olojede, et al., 2018). SBM is the byproduct of the oil extraction process, while RPM is the residual material left after the removal of starch. Both are exposed to heat treatment, which may influence AA digestibility. The AA digestibility can also be affected by several other factors like breed and age of the birds (Barua, et al., 2024). The possible reason for lower digestibility of RPM may point out the process of starch removal. Time and intensity of temperature enhance the process of starch removal, which possibly affects the quality of residues, particularly the protein (Wani, et al., 2018). Heating at around 90 to 95°C for a period of 2 h or more may affect the quality of protein and peptide linkage bonds in the raw material, especially the heat labile AA (Bandegan, et al., 2010)*.*

Sex of broiler birds has shown variable behavior in terms of nutrient utilization. Male broilers are dominant in terms of feed intake and in house activity (England, et al., 2023). In previous researches on broiler sex, the researchers revealed that the efficiency of lysine utilization for growth in male broilers is unaffected in the growth phase (Nogueira, et al., 2021). Male broilers develop their gut slightly earlier than female broilers due to faster feed intake and are capable of more efficient utilization of nutrients and AA, which are the building blocks for muscle protein and breast meat yield. The SID of arginine in this study was not affected by sex, confirming the outcome of previous study (Nogueira, et al., 2021)

In the present study, birds were given *ad-libitum* experimental diets for 7 days, and samples of digesta were collected on the 29th day of broiler age. Broiler age and adaptation period may affect the SID values (Barua, et al., 2024). Recent studies have demonstrated that digestibility of different AA in broilers increases with age during the first three weeks of bird's life (Barua, et al., 2021). Thus, basal endogenous losses also need to be standardized according to specific age (Ali, et al., 2023). The objective of adding protease was based on several AA digestibility trials, which have shown that exogenous protease supplementation increases ileal AA digestibility in broilers (Silva, et al., 2022). The overall impact on nutrient digestibility was non-significant with the added protease in our study. Despite higher AA digestibility of rice grain (Honda, et al., 2011), the resulted residual product from rice starch industry did not meet the comparable AA digestible values of SBM. This emphasizes the need for a further fine-tunning of the starch removal processing techniques to minimize damage to the nutrient availability (Wijethunga, et al., 2024). Temperature required during the enzymatic application to remove maximum starch contents from the grain is the point of concern for final product quality. The processing of rice protein meal, such as high-temperature treatment and chemical modifications, can cause protein denaturation and the formation of Maillard reaction products. These structural changes reduce protease accessibility, limiting its ability to enhance amino acid digestibility in broilers. By applying alternative techniques for starch removal process will help to rectify the lower digestibility issues in RPM. The current economic situation has forced many non-soybean producing countries to fully exploit the true nutritional potential of locally-produced, unconventional feed stuffs in poultry feed (Perttilä, et al., 2002).

## Authors’ Contributions

Nasir Mukhtar structur the experimental trial and supervised each step of the research while serving as the student supervisor and reviewed the manuscript. Muhammad Asad Iqbal executed the entire trial, prepared the original draft, and conducted the statistical analysis of the data. Tanveer Ahmad supervised the research trial, reviewed the draft and helped the technical inputs in designing the trial, while Usama Aftab also provided technical inputs in designing the trial and reviewed the final draft.

## Declaration of competing interest

The authors declare that they have no competing interests.
